# Opportunities for play in paediatric healthcare environments: a scoping review

**DOI:** 10.3389/fresc.2024.1415609

**Published:** 2024-05-30

**Authors:** Clarissa Yu, Sophie Weaver, Meaghan Walker, Julia Hess, Amanda Mac, Timothy Ross

**Affiliations:** ^1^ Bloorview Research Institute, Holland Bloorview Kids Rehabilitation Hospital, Toronto, ON, Canada; ^2^ Department of Geography & Planning, University of Toronto, Toronto, ON, Canada; ^3^ Rehabilitation Sciences Institute, University of Toronto, Toronto, ON, Canada

**Keywords:** play, play space, paediatric, healthcare, hospital, design, inclusion, accessibility

## Abstract

Play spaces are important components of paediatric healthcare environments. They provide children with critical opportunities to experience the social, emotional, and developmental benefits of play while in healthcare settings for appointments or hospitalizations. These spaces can help to mitigate stress, provide a sense of normalcy in unfamiliar environments, and facilitate social engagement for children and their families. Given the benefits of play spaces in paediatric healthcare settings, it is important to understand how these spaces can be designed to enhance children's inclusion and quality of care. The aim of this scoping review was to explore the current understanding of paediatric play space design. Using search terms related to children, health care, and play space, six interdisciplinary databases were searched over a 30-year period. The search found 2,533 records from which eighteen were included for review. Findings suggest that although it is well-documented that play spaces offer valuable social and emotional benefits, little is known about the specific design features that can and should be incorporated to enhance play opportunities and ensure that they benefit all children and families. Further, the literature mostly considers play spaces in the context of designated play or recreational rooms. Scholars are encouraged to consider how play opportunities can be incorporated into the designs of paediatric healthcare environments beyond the boundaries of these rooms. Future studies should also consider the diversity of play space users, including children of varying ages and abilities, to create more accessible and inclusive paediatric play spaces for children and their families. Advancing knowledge on play space design can help to optimize the quality of these important spaces and to ensure their designs meaningfully enhance children's play experiences and quality of care.

## 1 Introduction

Play, characterized as a self-directed activity in which children participate for fun, is an essential component of childhood (1, 2). It provides unique opportunities for children to explore their environments, interact and build relationships with peers, and take on new challenges (2, 3). Play experiences contribute to children's social, cognitive, and physical health, and help them to foster resilience and undergo healthy brain development (3, 4). Given its clear significance to children's wellbeing and development, it is important that all children are afforded ample opportunity to engage in play. The United Nations (1989) acknowledges the importance of children's play through its Convention on the Rights of the Child, which acknowledges play as a childhood right (5).

Unfortunately, not all children have equal opportunities to play; children living with disability and/or chronic illnesses often have fewer opportunities due to inaccessible designs, concerns associated with their disability or illness, and time spent undergoing frequent healthcare visits (2). As such, efforts have been made to integrate play opportunities into healthcare environments and the effects have thus far been positive. For example, healthcare providers use therapeutic play (i.e., organized activities designed to support children's psychosocial health) to enhance the delivery of care (6, 7). It has been shown that integrating therapeutic play into pre-procedural information sessions can mitigate children's anxiety before surgery and other invasive procedures (8–10). This is beneficial given that higher preoperative anxiety has been associated with more surgical and post-operative complications, longer inpatient stays, and sleep disturbances (11, 12). Further, children themselves have reported that play serves as a welcomed distraction from illness and helps them to cope while in hospital (13, 14). Play is also valued because it can provide children with a sense of agency in unfamiliar healthcare environments and can help to facilitate social engagement (15, 16). Play represents an increasingly important aspect of paediatric healthcare provision. Creating opportunities for play warrants critical attention among paediatric healthcare scholars and practitioners, as well as professionals involved in the design, building, and operation of paediatric healthcare spaces (e.g., planners, designers, architects, operations staff).

To integrate play into paediatric (and adult) healthcare settings such that it enhances quality of care, it is necessary to understand what factors affect children's ability to play and their experiences of play in healthcare settings. One important but frequently overlooked factor is the design of paediatric play spaces. It is well documented that the design of healthcare built environments (i.e., the physical architecture, interior design elements, and ambiance of a space) affects the ways in which care is experienced (15, 17–20). Incorporating inviting colours, biophilic elements, natural lighting, and private spaces can help to cultivate a therapeutic environment that can comfort children and their families, and support recoveries and well-being (19). Similarly, the design of paediatric play spaces can influence children's ability and desire to engage in play (15, 21). For example, the absence of a playroom in paediatric healthcare settings can serve as a barrier to play and interaction for children, whereas including playrooms with flexible seating options and play opportunities at accessible tables can help to ensure that children using mobility devices can easily engage in play activities (21). Despite the importance and benefits of offering physical play spaces in children's healthcare settings, little is known about how we can optimize the design and operation of these play spaces to advance inclusive play opportunities for children. To help address this knowledge gap, we conducted a review that engages the question, “what does the literature tell us about the design of play spaces in paediatric healthcare environments?” To engage this broad question, we opted to use a scoping review approach, as it is practical for identifying studies, mapping out the available evidence, and providing an overview of the topics that the identified studies have considered (22). The aim of this review is to provide a knowledge foundation for playspace design in paediatric healthcare environments that can be used to inform and guide future research on this topic.

## 2 Methods

Our review methodology followed Arksey and O'Malley's (23) scoping review approach. They recommend carrying out five stages to conduct a rigorous scoping review: (1) Identifying the research question; (2) Identifying relevant studies; (3) Study selection; (4) Charting the data; and (5) Collating, summarizing and reporting the results. The following subsections explain how we carried out each stage.

### 2.1 Identifying the research question

Our team's initial research question was specific in scope and presented challenges for capturing a breadth of coverage. Through team discussion and consultation with a health sciences librarian and the scoping/systematic review methodology literature (i.e., Munn et al, 2018; Levac et al., 2010; Arksey & O'Malley, 2005), the question was broadened so that the review would generate greater breadth of coverage (22–24). The broadened question (“what does the literature tell us about the design of play spaces in paediatric healthcare environments?”) aligned with our aim to produce a review that offers foundational knowledge for play space design in paediatric healthcare environments that can support future research.

### 2.2 Identifying relevant studies

To identify relevant studies, we developed a comprehensive search strategy. Six literature databases covering both health science and interdisciplinary topics were identified for searching. They were (1) Medline via OVID, (2) PsycInfo via OVID, (3) CINAHL, (4) Web of Science, (5) Scopus, and (6) Sociological Abstracts. The databases were selected because of their potential relevance to the intersecting topics associated with this review: paediatric healthcare, play, and architectural/spatial design. A 30-year period (i.e., 1991–2021) was applied to all database searches. This search period was selected because it aligns with the implementation of the 1991 *Americans with Disabilities Act—*a landmark piece of accessibility legislation that has had international influence on incorporating disability and accessibility considerations into the planning and design of built environments. Each database was searched using a comprehensive search strategy and article records underwent a rigorous screening process to identify the articles included in this review.

Working in consultation with a health sciences librarian, we developed a search string that was applied to each of the selected databases. The search string comprised terms associated with three topics relevant to this review: (1) children, (2) healthcare, and (3) play space. The terms were combined using a Boolean “AND” operator. To increase the breadth of the search, relevant terms and concepts related to each topic were included and connected via a Boolean “OR” operator. A wildcard function (?) was used to capture terms with different spellings (e.g., pediatric and paediatric) and a truncation function (*) was applied to capture different endings of root words (e.g., “teen*” was used to capture “teen”, “teens”, “teenager”, “teenagers”, etc.). The baseline search string was then modified to suit syntax requirements of each database to search the titles, abstracts, and keywords of records. The final search string is presented below:

(1) “p?ediatric” OR “youth” OR “kid*” OR “child*” OR “teen*” OR “adolescen*” OR “young person” OR “young people” OR “juvenile”
(2) AND “health care” OR “healthcare” OR “medicine” OR “rehab*” OR “hospital*” OR “clinic*” OR “medical” OR “treatment” OR “patient care” OR “infirmar*” OR “sanatorium*” OR “primary care” OR “urgent care” OR “emergency room” OR “doctor* office*”
(3) AND “play space*” OR “playspace*” OR “play room*” OR “playroom*” OR “play place*” OR “play area*” OR “play park*” OR “playground*” OR “jungle gym*” OR “therapeutic play” OR “healing space*” OR “play equipment” OR “recreation* ground*” OR “play garden*” OR “hospital garden*” OR “therap* garden*” OR “healing garden*” OR “sensory garden*” OR “game* room*” OR “rec room*” OR “recreation room*” OR “social space*” OR “leisure space*” OR “play environment*”

### 2.3 Study selection

Database searches were conducted in June 2021 and yielded 2,533 records. Records were uploaded into a database on Covidence.org, which offers a user-friendly interface for literature review screening processes. The Covidence software removed 947 duplicate records, leaving a total of 1,586 records for screening. Three reviewers (SW, AM, JH) screened the titles and abstracts of these records by applying the following inclusion criteria:

(A) Published between 1991 and 2021;
(B) Full-text available in English;
(C) Peer-reviewed, empirical research;
(D) Focused on children aged 0–18, their families, and/or clinicians;
(E) Described experiences in paediatric healthcare play spaces.

Articles were excluded if they met these exclusion criteria:

(A) Dissertation or thesis;
(B) Conference proceeding;
(C) Commentary or position statement;
(D) Literature review.

Each record was reviewed twice (i.e., once by two different reviewers). Two independent “yes” votes were required for the article to proceed to the full-text screening stage. The title and abstract screening process resulted in the exclusion of 1,541 records, leaving 45 records for full-text screening. Full-text screening also required two “yes” votes from two different reviewers. Of the 45 full texts assessed, 31 were excluded and 14 were included. When screening conflicts arose, two reviewers (SW, JH) discussed the study in question and consulted a senior researcher (TR) when help was needed to reach consensus.

**Figure 1:**
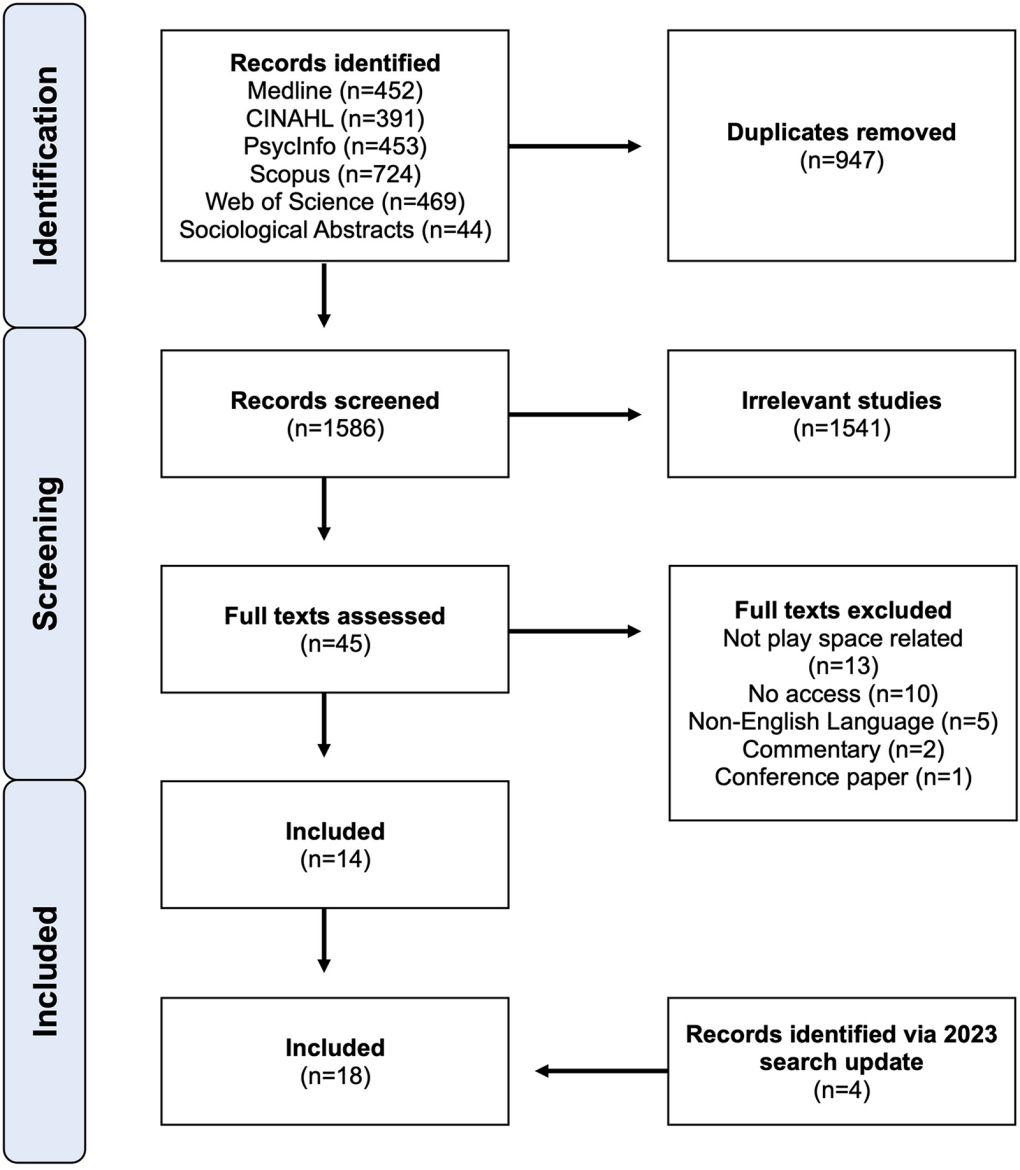
PRISMA diagram outlining screening process and record totals.

To ensure this review captured the most up-to-date literature, database searches were repeated in June 2023, and were applied to a two-year period from June 2021–May 2023. This updated search identified 335 records, from which 134 duplicates were removed, leaving 201 records for review. We repeated the same screening process using the same criteria. This yielded an additional 4 studies (25–28) for inclusion in this review. This brought the total number of articles included in this review to 18. Figure 1 outlines the screening process and record totals for this scoping review.

### 2.4 Charting the data

To chart the data, the research team discussed the relevant information that needed to be extracted from each article. This included the following: author, publication date, study population, location of research, study aim, methods used, and play design-related findings. This information was selected for extraction and charting because it was relevant to the research question (e.g., play-related findings) and/or because it could help to identify issues and gaps in the current literature (e.g., location of study could offer insight into geographic gaps). To extract data, each study was read in entirety and relevant data was extracted and charted into a table using Microsoft Excel software (see Table 1). Two independent reviewers extracted data from each study to ensure consistency.

**Table 1:** Summary of reviewed studies.

Study	Population & Location	Description	Play-Related Findings
1. Cunningham et al., (29)	Population: 290 healthcare staff, 177 clients and community organizations who work with children and families	Aim: To segment healthcare centre users and describe each group’s unique building design preferences to inform the design of a children’s health centre.	Authors identified three distinct user segments, each with unique healthcare built environment preferences. Access to play spaces (e.g., outdoor playgrounds, indoor gyms) was important to two segments.
	Location: Paediatric health centre, Canada	Methods: Discrete choice experiment survey developed from economic and marketing research	
2. Ebrahimpour et al., (25)	Population: 20 children hospitalized with cancer, aged 6–12	Aim: To identify and describe features of an oncology ward that symbolize hope for hospitalized children.	Playrooms distract from illness, boredom, and provide opportunities for socialization in hospital. Playrooms can symbolize hope in the oncology ward.
	Location: Paediatric hospital oncology ward, Iran	Methods: Photovoice, semi-structured interviews	
3. Ferreira et al., (30)	Population: 12 hospitalized children, aged 6–11	Aim: To describe children’s perspectives on the meaning of play in hospital, and the ability of the hospital environment to support play.	Children in hospital associate play with playrooms and report few opportunities for play beyond these spaces. Playrooms provide a safe, peaceful, and homelike environment that fosters social engagement and resilience.
	Location: Hospital’s paediatric ward, Brazil		
		Methods: Drawing, observations	
4. Franco Da Silva et al., (31)	Population: 9 children hospitalized with acute or chronic illness, average age 9	Aim: To describe children’s perceptions of and preferences for play as a component of care.	Play spaces facilitate coping and support children’s psychosocial health while in hospital. Children value age-appropriate opportunities for self-directed and guided play.
	Location: Hospital’s paediatric ward playroom, Brazil	Methods: Observations, art activities, interviews	
5. Hayhoe et al., (32)	Population: 68 children undergoing surgery, aged 1–11	Aim: To determine the effect of a preoperative anxiety-reduction play-related program on post-operative distress.	An anxiety reduction program with a preoperative play space was reported to help reduce children’s distress post-operatively when waking from anesthesia.
	Location: Paediatric hospital day surgical unit, United Kingdom		
		Methods: Prospective observational, Yale Preoperative Anxiety Scale	
6. Henderson-Wilson et al., (26)	Population: 19 staff, 36 parents	Aim: To describe how garden users benefit from a paediatric healthcare centre’s sensory garden.	The garden provided new play opportunities and encouraged outdoor play. Parents and staff reported that the garden promoted sensory play.
	Location: Paediatric healthcare centre’s sensory garden, Australia		
		Methods: Surveys, interviews	
7. Hosseinpour & Memarzadeh, (33)	Population: 200 children undergoing surgery, average ages 3–4	Aim: To determine whether waiting for elective surgery in playrooms can reduce children’s preoperative anxiety.	Waiting in playrooms significantly reduces preoperative anxiety vs. traditional waiting rooms. Children in playrooms are more active and demonstrate less distress and parental reliance.
	Location: Paediatric hospital surgical unit, Iran	Methods: Randomized controlled trial, Yale Preoperative Anxiety Scale	
8. Kelada et al., (27)	Population: 123 parents of hospitalised children	Aim: To describe how families use, experience, and perceive recreational rooms in a paediatric hospital.	Playrooms are generally associated with positive emotions. Parents value playrooms for the escape and social support they offer. The rooms can be difficult to find and lack activities for all.
	Location: Paediatric hospital, Australia		
		Methods: Questionnaire, interviews	
9. Lambert et al., (34)	Population: 55 children hospitalized with acute or chronic illness, aged 5–8	Aim: To identify and describe children’s preferences for social spaces in hospital.	Children prefer age- and gender-appropriate play opportunities integrated throughout the hospital. Play spaces facilitate valued social engagement.
		Methods: Semi-structured interviews with art activities	
	Location: 3 paediatric hospitals, Ireland		
10. Larsen & Agerskov, (28)	Population: 10 parents of children aged 6–11 hospitalized with epilepsy	Aim: To describe how parents of hospitalized children with epilepsy experience a hospital playground.	The playground helped families cope with hospitalization by providing distractions and opportunities for socialization. Playgrounds offer children a sense of normalcy, control, and a safe space to communicate and learn.
	Location: Playground in epilepsy hospital, Denmark	Methods: Semi-structured interviews	
11. McKinty, (35)	Population: children, their families, staff	Aim: To describe a pilot project that introduced children’s traditional play culture (e.g., classic games) into a paediatric hospital and its impacts on clients and staff.	Experiences with the play space and activities were positive. Play provided in-hospital distractions, reprieves, and feelings of normalcy. Traditional games fostered intergenerational connections among children, families, and staff.
	Location: Paediatric hospital, Australia		
		Methods: Analysis of prior observations and interviews	
12. Pasha, (36)	Population: 70 staff, 76 family members	Aim: To identify which design elements encourage and detour garden use in paediatric hospitals.	One reason parents visit hospital gardens is to let children play. Increasing play opportunities in the garden was recommended to enhance user experiences.
	Location: 5 gardens in 3 paediatric hospitals, United States		
		Methods: Questionnaires (structured, open-ended questions)	
13. Pasha & Shepley, (37)	Population: 82 staff, 49 adult family members, 53 children	Aim: To determine the correlation between garden design features and user physical activity in paediatric hospital gardens.	Users engage in different levels of physical activity based on garden design. Gardens with unique pathways, layouts, and child-oriented play and seating elements had greater user physical activity.
	Location: 5 gardens in 3 paediatric hospitals, United States		
		Methods: Observations, surveys, Children Hospital Garden Audit Tool	
14. Reeve et al., (38)	Population: patients, family members, staff	Aim: To describe how garden users experience, perceive, and benefit from two paediatric garden spaces.	Gardens are viewed as places to relax, reflect, enjoy nature, and play. Parents value the escape they offer to children.
	Location: Paediatric hospital garden, Australia		
		Methods: Thematic analysis of visitor book comments	
15. Sherman et al., (39)	Population: 1,400 garden users (patients, families, staff, visitors)	Aim: To describe the utilization patterns of three paediatric cancer center’s gardens.	Adults use the garden more than children despite its child-centred design. Children engage with garden elements (e.g., play features, sculptures) more than adults who use the space to relax. Users experience less distress in gardens vs. in hospital.
	Location: 3 paediatric cancer centre gardens, United States		
		Methods: Observations, surveys	
16. Turner et al., (40)	Population: 28 parents, 21 staff, 3 visitors	Aim: To describe how a play garden is perceived and experienced by adults and children from their parents’ perspective.	Adults enjoy the outdoor atmospheres of gardens. Parents value the garden for the escape, sense of normalcy, and structured and self-directed play opportunities it provides for children.
	Location: Play garden in a paediatric and women’s healthcare centre, Canada		
		Methods: Surveys (rating scales, open-ended questions)	
17. Weinberger et al., (41)	Population: 90 child life specialists, aged 22–62	Aim: To identify and describe what design features optimize a hospital playroom according to child life specialists and their therapeutic goals.	Nature elements, aesthetic colours, and open space were viewed as valuable features. Including many play options and age-appropriate space is important. A positive playroom environment enables meaningful engagement to support child life goals.
	Location: 5 paediatric hospital playrooms, United States		
		Methods: Surveys (Likert scale, open-ended questions)	
18. Whitehouse et al., (34)	Population: 16 paediatric patients, 6 siblings, 83 adults (staff and family members)	Aim: To conduct a post-occupancy evaluation assessing whether the garden mitigates stress, improves user satisfaction, and provides hope.	Staff and parents find that hospital gardens support their relaxation and encourage adding play opportunities into them. Hospitalized children use the garden less than their siblings.
	Location: Paediatric hospital gardens, United States		
		Methods: Observations, surveys, semi-structured interviews	

### 2.5 Collating, summarizing and reporting the results

Our work to collate, summarize, and report results involved producing a basic numerical analysis of the identified studies with respect to their publication date, geographic location, study population considered, types of methods used, and what play spaces were considered (see Results below). Our team also engaged in in-depth discussion about the reviewed articles and Table 1 to identify what themes in the literature warranted discussion. At the same time, we considered what knowledge gaps and weaknesses existed in the literature, and potential directions for future research.

## 3 Results

Table 1 presents a summary of the 18 studies included in this review. This table outlines each study's population, location, aim, methods, and play-related findings. Most of the reviewed studies were published after 2010 (*n* = 15), which suggests that scholarly attention to play environments in paediatric health care settings is a relatively recent development. Most studies were geographically situated in North America [United States (*n* = 5), Canada (*n* = 2)] and Australia (*n* = 4), which suggests that there are knowledge gaps across (and lessons to be learned from) other geographies. In particular, little research has emerged from developing nations, indicating that there is a notable need for studies focused on play spaces within paediatric health care environments within these nations. The remaining studies were based in Brazil (*n* = 2), Iran (*n* = 2), Denmark (*n* = 1), Ireland (*n* = 1), and the United Kingdom (*n* = 1).

Most studies focused on the experiences of children hospitalized for acute or chronic illnesses, and/or their parents. Studies either focused exclusively on the experiences of adult family members (*n* = 2), healthcare professionals (*n* = 1), children (*n* = 6), or some combination of these groups (*n* = 9). Of the 6 studies focusing exclusively on children, all children were aged 12 or under. None of the studies indicated whether children with disabilities and their families were included in study populations. The experiences and preferences of these individuals across all studies were gathered using qualitative (*n* = 7), quantitative (*n* = 4), or mixed methods (*n* = 7). Examples of common qualitative methods include arts-based activities (25, 30, 31, 34), observations (30, 31, 35, 37, 39, 42), and interviews (25–28, 31, 34, 35, 42), whereas quantitative studies mostly used validated questionnaires (32, 33, 37) and surveys (26, 27, 29, 36, 37, 39–42).

Two thirds of the reviewed studies (*n* = 12) reported on specific types of play spaces in hospital settings, including gardens (*n* = 7), designated play or recreational rooms (*n* = 4), and playgrounds (*n* = 1). The remaining third of studies (*n* = 6) took a more generalized approach and reported on the experiences of play spaces without focusing on a specific location. No studies discussed the incorporation of accessible or inclusive designs that account for the presence and diversity of childhood disability.

## 4 Discussion

In the following subsections, we discuss three key themes that emerged from our review of the literature. These themes are: (1) Emotional and Social Benefits of Play Spaces in Paediatric Health Care Environments; (2) Paediatric Play Space Design Needs and Preferences; and (3) Unsettling and Expanding the Boundaries of Play. Following discussion of these themes, we identify potential future research directions that require scholarly attention to advance thinking about inclusive play design within paediatric healthcare environments.

### 4.1 Emotional and social benefits of play spaces in paediatric health care environments

To appreciate the value of play spaces in paediatric health care settings, it is important to understand the benefits that they offer. The reviewed studies demonstrate that paediatric healthcare play spaces offer a wide range of social and emotional benefits to both children and their families. First, these spaces are valued for facilitating social engagement, as they create meaningful social opportunities that can enable both children and parents to connect with others who may share experiences of illness and hospitalization (25, 27, 28, 30, 31, 34, 35). One study noted that play spaces help to foster community and intergenerational connections within healthcare settings (35). Aside from their social function, play spaces are also valued because they can serve as an escape or distraction from illness, boredom, and hospitalization (25). Play spaces in children's healthcare settings are typically viewed as safe, homelike spaces that provide children with a sense of agency and normalcy within the broader (and, arguably colder or less child-friendly) institutional healthcare environment (25, 27, 28, 35, 38, 40, 42). Further, play spaces provide places to relax and relieve stress, which is beneficial to the psychosocial health of both children and their family members (27, 31–33, 38, 39, 42). The reviewed studies demonstrate a wide range of social and emotional benefits that play spaces in paediatric healthcare settings can offer to children and their families; in turn, play spaces can contribute to the enhancement of their paediatric care experiences (15, 18).

### 4.2 Paediatric play space design needs and preferences

Given the clear social and emotional benefits of play spaces in paediatric healthcare settings, it follows that these spaces should be designed to enable children and their families to experience their benefits such that they enhance care. Designing spaces with the intent to optimize user experiences describes the practice of evidence-based design (EBD), which is well-recognized for its role in helping to optimize the design of healthcare environments based on empirical evidence (15, 43). EBD has been considered in the design of hospital wards, inpatient rooms, and clinical areas (43), and could be used to inform the design of paediatric play spaces. Applying EBD to play space design may help to ensure that these spaces are effectively designed to support intended usages by children with varying abilities and interests, their family members, and even staff (e.g., for play-based rehabilitation/therapeutic activities), such that all can leverage these spaces with a view to enjoy the social and emotional benefits of play (44). However, the 18 reviewed studies presented limited information about how paediatric healthcare play spaces and built environments in general can be designed to support play. That is, little attention has been given to understanding what specific design features (e.g., floorplan, décor, colours, lighting, and play elements) should be prioritized to ensure that play spaces can provide children with opportunities to experience the emotional and social benefits associated with play, such as a sense of normalcy, agency, and opportunities for social engagement. A knowledge gap persists regarding how the built environment can enhance play spaces, improve user experiences, and provide benefits to children and families within paediatric healthcare settings.

In questioning what specific built environment features should be prioritized to optimize user experiences with paediatric healthcare play spaces, it is important to know whose needs are being considered. Of the reviewed studies, more than half (11 of 18) included children as participants. Five of these studies included children in addition to adult family members and healthcare staff (35, 37–39, 42). The other six studies focused exclusively on children's experiences with paediatric play spaces (25, 30–34). However, a closer examination suggests that the perspectives of adolescents aged 12–18 are missing, as all child participants in the six studies focusing exclusively on children were aged 12 or younger. This is troubling as adolescents and younger children have different healthcare needs and experiences (20); for example, studies have found that these two groups' preferences for interior design and privacy concerns differ (21, 45). In fact, some adolescents have reported that they perceive most healthcare spaces are designed for younger children with little space to accommodate them (14, 46). Beyond having different healthcare needs and preferences, these two groups also have different needs, preferences, and attitudes toward play. In paediatric settings, adolescents have indicated that play spaces and activities are typically designed to engage younger children and that there are fewer opportunities for adolescent recreation (46). The literature on play in paediatric healthcare settings has largely focused on play space experiences as they relate to children under 12 years of age and has overlooked the play needs and preferences of adolescents. This has occurred even though adolescents receive care in the same settings, and they can also enjoy the social and emotional benefits of play and recreation (2, 47). Given that some adolescents with chronic illnesses may visit healthcare settings frequently, and as a result, miss out on opportunities for socialization elsewhere, it would be beneficial if paediatric healthcare centres were to create play/recreation spaces that are designed with their needs and preferences in mind (2). Scholars should be encouraged to include greater adolescent representation in future research on this topic to help advance age-appropriate play space designs that help to optimize healthcare experiences for all children and youth.

### 4.3 Unsettling and expanding the boundaries of play

The third key theme that emerged from the literature concerns what spaces we do and do not perceive as play spaces in paediatric healthcare settings. The literature mostly considers play spaces in the context of designated play and recreational rooms, playgrounds, or gardens. Two thirds of the reviewed studies (12 of 18) focused on the experiences and preferences of children, their families, and/or staff in relation to these specific play spaces (27, 28, 33, 35–42, 48). Little scholarly attention has been given to the possibility of incorporating play elements or spaces beyond these specific settings and evidence suggests that there are limited play opportunities beyond designated play spaces. For example, Ferreira et al. (2014) found that while children valued the play opportunities that designated playrooms present, they perceived few opportunities for play within the broader hospital setting (30). This finding, combined with the literature's general inattention to play beyond the boundaries of designated play spaces, suggests that current paediatric healthcare environment designs tend to isolate play to certain spaces. However, this design practice does not align with children's play preferences in healthcare settings. Lambert et al. (2014) found that although children value designated play spaces, they would like to have play opportunities incorporated throughout hospital spaces, including at their bedside and in inpatient rooms (34). Rollins (2009) similarly reported that spaces other than the conventional playrooms were being used for play activities (21). In fact, children were observed using hallways to play games, and play was made available at the bedside for those unable to visit playrooms (21). Findings indicate that there is a need to question and expand upon what spaces can present play opportunities within paediatric healthcare settings, which could present unique and exciting opportunities for healthcare built environment designers and administrators to enhance care experiences through more thoughtful and playful spatial designs that extend beyond the boundaries of designated play spaces.

One strategy that could be used to enhance the diversity of play opportunities throughout paediatric healthcare stings is through technology. There has been significant interest in leveraging interactive technology to enhance children's care experiences (15, 34, 49–51). Sermon (2007) created an interactive art installation in the waiting space of a paediatric hospital, which was intended to mitigate anxiety and create an enjoyable and therapeutic waiting experience (51). Initial staff feedback indicated that the installation was successful in providing a positive distraction for waiting children (51). Similarly, Biddiss et al. (2013) developed ScreenPlay, an interactive and inclusive hands-free technology that can be incorporated into waiting rooms so children can play games while waiting (49). In the study, both children and adults had positive experiences, children's reported anxiety levels were lower with ScreenPlay, and the addition of ScreenPlay increased user satisfaction with the waiting space (49, 52). Other studies describe similar benefits of incorporating interactive technologies into healthcare settings to enhance user experiences (15, 50). The literature indicates that incorporating interactive technologies oriented toward creating new play opportunities within paediatric healthcare spaces can enhance the care experiences of children and their families. Such technologies could be further leveraged to increase the play opportunities available in healthcare spaces (e.g., inpatient rooms, atria, hallways, and outdoor spaces).

### 4.4 Future directions: designing for accessible and inclusive play

Inattention to disability and the need for accessible and inclusive play designs represents another knowledge gap in the literature intersecting play and paediatric healthcare. Most of the reviewed studies reported on play space experiences of children and their families in general without exploring the specific experiences of those living with childhood disability. Attention to their specific play experiences is warranted given that they have been found to visit healthcare settings more often than their peers without disabilities (53). Further, children with disabilities may have different mobility and/or sensory needs (and thus different accessibility requirements) than their peers (54, 55). If play spaces are not designed with the accessibility and inclusion of children with disabilities in mind, these spaces may yield negative experiences of exclusion from play opportunities and cause these children to miss out on the benefits of play. For example, Whitehouse et al. (2001) observed that few children with severe chronic illnesses and disability used the paediatric hospital garden, concluding that these children may face unique needs that have not been accommodated in the space (42). Their observation highlights the importance of accounting for the presence and diversity of childhood disability in the designs of paediatric play spaces (42).

The concept of inclusive play design is geared toward identifying and dismantling barriers to play, which in turn creates opportunities for children with and without disabilities to experience the benefits of play in a safe environment (1). In recent years, inclusive play designs have been incorporated into many playgrounds, where studies and design resources have identified an array of design features (e.g., play surface materials, play equipment, pathways) that should be considered to ensure inclusive play spaces (3, 56–58). Inclusive play can and should be considered for the design of play spaces in health care settings similar to how it has been considered for the design of playgrounds. We note that it should be considered given the heightened presence of children with disabilities in healthcare contexts and the fact that inclusive playground design practices (see Ross et al., 2022) (57) could enhance and increase the number of play opportunities in healthcare settings. Thinking about inclusive play when designing healthcare play spaces, and healthcare environments in general, could provide healthcare environment designers, planners, and builders with valuable knowledge concerning what design features should be prioritized to enhance the inclusivity of play spaces such that all children can engage in play and enjoy its social and emotional benefits while receiving care.

## 5 Conclusion

In this scoping review we have considered the findings of 18 studies that examined how children, their families, and staff experience play spaces within paediatric healthcare environments. The reviewed literature documents significant social and emotional benefits associated with play in healthcare settings, which can help to encourage the increased presence of play opportunities throughout paediatric healthcare environments. The included studies also show how users value and use play spaces, offer insight into the perceived realm of play in healthcare settings, and demonstrate gaps in understanding regarding play space design. Further research is needed to understand what specific design elements can enhance play spaces and play opportunities for children. Scholars carrying out such research should be encouraged to account for the play needs and preferences of adolescents whose paediatric healthcare play experiences and preferences have gone largely overlooked to date. Scholars should also be encouraged to question how we can move beyond current perceptions of where play can and cannot occur in paediatric healthcare settings, as this may help with incorporating desired play opportunities into spaces that are beyond the boundaries of typical designated play spaces. For example, it could help with creating play opportunities within inpatient rooms, atria, hallways, and outdoor spaces; in turn, this could extend the benefits of play into new spaces in healthcare settings. Finally, future studies on play in paediatric healthcare settings must consider the accessibility needs and inclusion of children with disabilities to help create more equitable access to play opportunities for them. Exploring these research avenues will help to advance the current understanding of play space design in paediatric healthcare settings. Advancing knowledge on this topic can help to optimize the design of play spaces such that they can enhance play experiences and the quality of care.

## References

[1] Brown DMY Ross T Leo J Buliung RN Shirazipour CH Latimer-Cheung AE A scoping review of evidence-informed recommendations for designing inclusive playgrounds. Front Rehabil Sci. (2021) 2:664595. 10.3389/fresc.2021.664595 36188796 PMC9397725

[2] Nijhof SL Vinkers CH van Geelen SM Duijff SN Achterberg EJM van der Net J Healthy play, better coping: the importance of play for the development of children in health and disease. Neurosci Biobehav Rev. (2018) 95:421–9. 10.1016/j.neubiorev.2018.09.024 30273634

[3] Ginsburg KR. American academy of pediatrics committee on communications, American academy of pediatrics committee on psychosocial aspects of child and family health. The importance of play in promoting healthy child development and maintaining strong parent-child bonds. Pediatrics. (2007) 119(1):182–91. 10.1542/peds.2006-2697 17200287

[4] Yogman M Garner A Hutchinson J Hirsh-Pasek K Golinkoff RM, Committee on psychosocial aspects of child and family health, et al. The power of play: a pediatric role in enhancing development in young children. Pediatrics. (2018) 142(3). 10.1542/peds.2018-2058 30126932

[5] United Nations General Assembly. Convention on the rights of the child. U. N. Treaty Ser. (1989) 1577:3.

[6] Godino-Iáñez MJ Martos-Cabrera MB Suleiman-Martos N Gómez-Urquiza JL Vargas-Román K Membrive-Jiménez MJ Play therapy as an intervention in hospitalized children: a systematic review. Healthcare. (2020) 8(3):239. 10.3390/healthcare8030239 32751225 PMC7551498

[7] Wong CL Ip WY Kwok BMC Choi KC Ng BKW Chan CWH. Effects of therapeutic play on children undergoing cast-removal procedures: a randomised controlled trial. BMJ Open. (2018) 8(7):e021071. 10.1136/bmjopen-2017-021071 29980545 PMC6042539

[8] Halemani K Issac A Mishra P Dhiraaj S Mandelia A Mathias E. Effectiveness of preoperative therapeutic play on anxiety among children undergoing invasive procedure: a systematic review and meta-analysis. Indian J Surg Oncol. (2022) 13(4):858–67. 10.1007/s13193-022-01571-1 36687245 PMC9845488

[9] Li HCW Lopez V Lee TLI. Effects of preoperative therapeutic play on outcomes of school-age children undergoing day surgery. Res Nurs Health. (2007) 30(3):320–32. 10.1002/nur.20191 17514706

[10] Zengin M Yayan EH Düken ME. The effects of a therapeutic play/play therapy program on the fear and anxiety levels of hospitalized children after liver transplantation. J Perianesth Nurs. (2021) 36(1):81–5. 10.1016/j.jopan.2020.07.006 33158746

[11] Dehghan F Jalali R Bashiri H. The effect of virtual reality technology on preoperative anxiety in children: a solomon four-group randomized clinical trial. Perioper Med. (2019) 8(1):5. 10.1186/s13741-019-0116-0 PMC6549331 31171963

[12] Kain ZN Mayes LC Caldwell-Andrews AA Karas DE McClain BC. Preoperative anxiety, postoperative pain, and behavioral recovery in young children undergoing surgery. Pediatrics. (2006) 118(2):651–8. 10.1542/peds.2005-2920 16882820

[13] Boyd JR Hunsberger M. Chronically ill children coping with repeated hospitalizations: their perceptions and suggested interventions. J Pediatr Nurs. (1998) 13(6):330–42. 10.1016/S0882-5963(98)80021-3 9879169

[14] Gürcan M Atay Turan S. Examining the expectations of healing care environment of hospitalized children with cancer based on watson’s theory of human caring. J Adv Nurs. (2021) 77(8):3472–82. 10.1111/jan.14934 34142737

[15] Jiang S. Positive distractions and play in the public spaces of pediatric healthcare environments: a literature review. Health Environ Res Des J. (2020) 13(3):171–97. 10.1177/1937586720901707 32008400

[16] Koukourikos K Tzeha L Pantelidou P Tsaloglidou A. The importance of play during hospitalization of children. Mater Sociomed. (2015) 27(6):438–41. 10.5455/msm.2015.27.438-441 26889107 PMC4733554

[17] Adams A Theodore D Goldenberg E McLaren C McKeever P. Kids in the atrium: comparing architectural intentions and children’s experiences in a pediatric hospital lobby. Soc Sci Med. (2010) 70(5):658–67. 10.1016/j.socscimed.2009.10.049 19962223

[18] Babbu AH Haque M. A framework for the design of pediatric healthcare environment using the delphi technique. Ain Shams Eng J. (2023) 14(5):101975. 10.1016/j.asej.2022.101975

[19] Gaminiesfahani H Lozanovska M Tucker R. A scoping review of the impact on children of the built environment design characteristics of healing spaces. Health Environ Res Des J. (2020) 13(4):98–114. 10.1177/1937586720903845 32077757

[20] Yu C Wong E Gignac J Walker M Ross T. A scoping review of pediatric healthcare built environment experiences and preferences among children with disabilities and their families. Health Environ Res Des J. (2024) 17(2):309–25. 10.1177/19375867231218035 PMC11080387 38130020

[21] Rollins JA. The influence of two hospitals’ designs and policies on social interaction and privacy as coping factors for children with cancer and their families. J Pediatr Hematol Oncol Nurs. (2009) 26(6):340–53. 10.1177/1043454209339734 19687464

[22] Munn Z Peters MDJ Stern C Tufanaru C McArthur A Aromataris E. Systematic review or scoping review? Guidance for authors when choosing between a systematic or scoping review approach. BMC Med Res Methodol. (2018) 18(1):143. 10.1186/s12874-018-0611-x 30453902 PMC6245623

[23] Arksey H O’Malley L. Scoping studies: towards a methodological framework. Int J Soc Res Methodol. (2005) 8(1):19–32. 10.1080/1364557032000119616

[24] Levac D Colquhoun H O'Brien KK. Scoping studies: advancing the methodology. Implement Sci. (2010) 5:69. 10.1186/1748-5908-5-69 20854677 PMC2954944

[25] Ebrahimpour F Mirlashari J Hosseini ASS Zarani F Thorne S. Symbols of hope on pediatric oncology ward: children’s perspective using photovoice. J Pediatr Hematol Oncol Nurs. (2021) 38(6):385–98. 10.1177/10434542211041934 34541954

[26] Henderson-Wilson C Shaw A Weerasuriya R. Perceived benefits of accessing a children’s sensory garden in a healthcare setting. Aust Health Rev. (2022) 46(5):573–6. 10.1071/AH22123 36070896

[27] Kelada L Wakefield CE De Graves S Treadgold C Dumlao G Schaffer M Evaluation of an in-hospital recreation room for hospitalised children and their families. J Pediatr Nurs. (2021) 61:191–8. 10.1016/j.pedn.2021.05.017 34118590

[28] Larsen MS Agerskov H. The importance of an outdoor playground for children with epilepsy and their family during and after hospitalization: a qualitative study of parents’ experiences. J Pediatr Nurs. (2022) 66:e16–21. 10.1016/j.pedn.2022.07.001 35811185

[29] Cunningham CE Niccols A Rimas H Robicheau R Anderson C DeVries B. Using a discrete choice conjoint experiment to engage stakeholders in the design of an outpatient children’s health center. Health Environ Res Des J. (2017) 10(5):12–27. 10.1177/1937586716686350 28068858

[30] Ferreira NAS Esmeraldo JD Blake MDT Antão JdL Raimundo RD Abreu LC. Social representation of the hospital ludic: look of the child. J Hum Growth Dev. (2014) 24(2):188–94. 10.7322/jhgd.81171

[31] Franco da Silva JÍ Barreto Pereira J Duarte Coutinho SE Figueiredo de Sá França JR Cortez Costa de Oliveira I Pereira do Carmo A Playfulness as a strategy in caring for hospitalized children. Saúde Coletiva. (2020) 10(52):2210–21. 10.36489/saudecoletiva.2020v10i52p2210-2221

[32] Hayhoe S Pallett S Zani J Trott J. Reduction of postanesthetic pediatric distress: a coordinated approach. J Perianesth Nurs. (2018) 33(3):312–318.e1. 10.1016/j.jopan.2016.11.005 29784261

[33] Hosseinpour M Memarzadeh M. Use of a preoperative playroom to prepare children for surgery. Eur J Pediatr Surg. (2010) 20(06):408–11. 10.1055/s-0030-1265172 21058244

[34] Lambert V Coad J Hicks P Glacken M. Social spaces for young children in hospital. Child Care Health Dev. (2014) 40(2):195–204. 10.1111/cch.12016 23294129

[35] McKinty J. From playground to patient: reflections on a traditional games project in a paediatric hospital. Int J Play. (2013) 2(3):187–201. 10.1080/21594937.2013.852053

[36] Pasha S. Barriers to garden visitation in children’s hospitals. Health Environ Res Des J. (2013) 6(4):76–96. 10.1177/193758671300600405 24089182

[37] Pasha S Shepley MM. Research note: physical activity in pediatric healing gardens. Landsc Urban Plan. (2013) 118:53–8. 10.1016/j.landurbplan.2013.05.005

[38] Reeve A Nieberler-Walker K Desha C. Healing gardens in children’s hospitals: reflections on benefits, preferences and design from visitors’ books. Urban Urban Green. (2017) 26:48–56. 10.1016/j.ufug.2017.05.013

[39] Sherman SA Varni JW Ulrich RS Malcarne VL. Post-occupancy evaluation of healing gardens in a pediatric cancer center. Landsc Urban Plan. (2005) 73(2–3):167–83. 10.1016/j.landurbplan.2004.11.013

[40] Turner J Fralic J Newman-Bennett K Skinner L. Everybody needs a break! responses to a playgarden survey. Pediatr Nurs. (2009) 35(1):27–34. 19378571

[41] Weinberger N Butler AG McGee B Schumacher PA Brown RL. Child life specialists’ evaluation of hospital playroom design: a mixed method inquiry. J Inter Des. (2017) 42(2):71–91.

[42] Whitehouse S Varni JW Seid M Cooper-Marcus C Ensberg MJ Jacobs JR Evaluating a children’s hospital garden environment: utilization and consumer satisfaction. J Environ Psychol. (2001) 21(3):301–14. 10.1006/jevp.2001.0224

[43] Ulrich RS Zimring C Zhu X DuBose J Seo HB Choi YS A review of the research literature on evidence-based healthcare design. Health Environments Research & Design Journal. (2008) 1(3):61–125. 10.1177/193758670800100306 21161908

[44] MacAllister L Zimring C Ryherd E. Environmental variables that influence patient satisfaction: a review of the literature. Health Environ Res Des J. (2016) 10(1):155–69. 10.1177/1937586716660825 27492078

[45] Peeters K Jellema P Annemans M Heylighen A. How do adolescents affected by cancer experience a hospital environment? J Adolesc Young Adult Oncol. (2018) 7(4):488–92. 10.1089/jayao.2017.0116 29583076

[46] Miralles PM Ramón NC Valero SA. Adolescents with cancer and occupational deprivation in hospital settings: a qualitative study. Hong Kong J Occup Ther. (2016) 27(1):26–34. 10.1016/j.hkjot.2016.05.001 30186058 PMC6091995

[47] Johnston O Wildy H Shand J. Teenagers learn through play too: communicating high expectations through a playful learning approach. Aust Educ Res. (2023) 50:921–40. 10.1007/s13384-022-00534-3

[48] Henderson J. Humanity by design. Industrial designer patricia moore’s products focus on the rehabilitation needs of elders and of the “differently-abled.”. Interiors. (1994) 153(8):58–9. 10172110

[49] Biddiss E McPherson A Shea G McKeever P. The design and testing of interactive hospital spaces to meet the needs of waiting children. Health Environ Res Des J. (2013) 6(3):49–68. 10.1177/193758671300600305 23817906

[50] Chen AYC Bongers B Iedema R. Visual melodies interactive installation for creating a relaxing environment in a healthcare setting. Proceedings of the 21st Annual Conference of the Australian Computer-Human Interaction Special Interest Group: Design: Open 24/7. New York, NY, USA: ACM; 2009. p. 361–4.

[51] Sermon P. The teleporter zone: interactive media arts in the healthcare context. Leonardo. (2007) 40(5):426–31. 10.1162/leon.2007.40.5.426

[52] Biddiss E Knibbe TJ Fehlings D McKeever P Cohen A McPherson A. Interactive media as a tool for reducing waiting anxiety at paediatric rehabilitation hospitals: a randomized controlled trial. Dev Med Child Neurol. (2018) 60(6):602–10. 10.1111/dmcn.13646 29243805

[53] Kim J Stevens P Carbone PS Jones KB. Health care use and spending of pediatric patients with an intellectual or developmental disability. Med Care. (2020) 58(5):468–73. 10.1097/MLR.0000000000001293 31934953

[54] Oliver BE Nesbit RJ McCloy R Harvey K Dodd HF. Adventurous play for a healthy childhood: facilitators and barriers identified by parents in Britain. Soc Sci Med. (2023) 323:115828. 10.1016/j.socscimed.2023.115828 36931037

[55] Wood EB Halverson A Harrison G Rosenkranz A. Creating a sensory-friendly pediatric emergency department. J Emerg Nurs. (2019) 45(4):415–24. 10.1016/j.jen.2018.12.002 30679010

[56] James ME Jianopoulos E Ross T Buliung R Arbour-Nicitopoulos KP. Children’s usage of inclusive playgrounds: a naturalistic observation study of play. Int J Environ Res Public Health. (2022) 19(20):13648. 10.3390/ijerph192013648 36294228 PMC9602768

[57] Ross T Arbour-Nicitopoulos K Kanics IM Leo J. Creating Inclusive Playgrounds: A Playbook of Considerations and Strategies. Toronto: Holland Bloorview Kids Rehabilitation Hospital (2022).

[58] van Engelen L Ebbers M Boonzaaijer M Bolster EAM van der Put EAH Bloemen MAT. Barriers, facilitators and solutions for active inclusive play for children with a physical disability in The Netherlands: a qualitative study. BMC Pediatr. (2021) 21:369. 10.1186/s12887-021-02827-5 34454470 PMC8401178

